# Countries urged to take tough action to prevent NCDs

**DOI:** 10.2471/BLT.17.031017

**Published:** 2017-10-01

## Abstract

Uruguay is hosting the WHO Global Conference on Noncommunicable Diseases (NCDs) this month, from 18–20 October. Tabaré Vázquez, the President of Uruguay, tells the *Bulletin of the World Health Organization* about his country’s efforts to prevent and fight NCDs and why countries should step up the global response.

Q: Why did you study medicine and what drew you to oncology?

A: As a child I always admired the family doctor who visited our family when one of us was sick. He brought with him a magic solution – or so it seemed – to our problems. From then on, I wanted to become a doctor. Later I decided to specialize in oncology after both my parents and my sister died of cancer in the 1960s.

Q: How did you become interested in public health?

A: In Uruguay, physicians train in public hospitals and university hospitals. These institutions open their doors to the poorest and most vulnerable people in society and, as junior doctors, we learn more about how poor people live. It was this experience that drew me to public health practice, and later, a public health policy that strives to improve the quality of health care and patients’ quality of life. Also, public health topics, including social medicine and epidemiology, are an important part of our medical training in Uruguay.

Q: What were your main public health initiatives as mayor of Montevideo?

A: When I took over as mayor of Montevideo, our country was going through a major economic crisis and there were several priorities. Many people could no longer afford health care and several private health-care centres closed. High unemployment was also a serious problem. Our response was to expand and strengthen primary care polyclinics across the city to meet the basic health needs of the population, in addition to providing housing, transportation and food. We decentralized health care by opening primary health care centres on the outskirts of the city, as these areas were poorly served, and we started providing child benefit.

Q: What were the most important health policies during your first term as president?

A: When I first came to office in 2005, we introduced several social reforms to reduce inequities. We immediately started moving towards universal health coverage (UHC), so that everyone in our country could access essential health services regardless of their ability to pay. We had just been through a severe economic crisis, huge disparities had developed between rich and poor. The health-care sector was fragmented, with many private and public providers of health care. In 2007, we launched the National Integrated Health System (*Sistema Nacional Integrado de Salud*) bringing together all the private and public sub-systems under one umbrella, to provide comprehensive and equitable health coverage for all. Our strategy has also been to shift health-care delivery towards primary health care, the prevention of noncommunicable diseases (NCDs) – as these cause by far the greatest burden of disease and death in Uruguay – and health promotion activities. That’s why Uruguay was one of the first countries to sign up to the World Health Organization (WHO) Framework Convention on Tobacco Control in 2003.

Q: Your tough tobacco control measures have made headlines around the world since then. Can you tell us about these measures?

A: Uruguay was the first country in Latin America to enact 100% smoke-free national legislation in 2006 by banning smoking in all indoor public places. By 2010, we had significantly increased the price and taxes on tobacco products and imposed an indoor smoking ban. We became the first country in the world to require that health warnings and striking images of the effects of tobacco are displayed on 80% of the principal surfaces of cigarette packages. We have banned tobacco advertising and misleading marketing descriptions, such as “light” and “ultralight” cigarettes because all of these products contain carcinogens and are harmful to people’s health. We banned the marketing of electronic cigarettes and we offer smoking cessation therapy for those who want to quit smoking. In 2012, we launched a public awareness campaign on the risks of smoking for women – especially during pregnancy. In 2014, we made the ban on tobacco advertising, promotion and sponsorship comprehensive. This ban includes the display of tobacco products at points of sale, digital and social media marketing and product placement in videos, film and television. All of these measures were taken for the simple reason that smoking kills.

Q: What are the results of these measures so far?

A: First, indoor air pollution has been reduced by 90%, according to a study looking at the change since smoke-free legislation was enacted. According to Yale University’s environmental performance index, Uruguay ranks first in the world in terms of the best indoor air quality. We reduced the prevalence of smoking in adults: from 32% in 2006 to 22.2% in 2014 and in young people aged 13–15 years from 23.2% in 2007 to 12.8% in 2014. In addition, after we passed 100% smoke-free legislation, we saw a 22% reduction in hospital admissions for acute myocardial infarction, in studies done one and two years after that. At the same time, while fewer people are smoking and tobacco sales have fallen, thanks to increases in tobacco tax rates, the state collected significantly more tobacco tax revenue: in 2004, US$ 84 million compared with US$ 318 million in 2011. There’s still a lot to do, but we are making progress.

Q: You mentioned strong preventive policies to reduce one important risk factor, but what about the detection and treatment of cancer?

A: In Uruguay, cancer has been the second leading cause of death after cardiovascular diseases for decades, accounting for more than 8000 deaths a year and about 25% of all deaths in our country. Our current national health plan includes several goals for cancer, including primary prevention of cancer, and timely detection and treatment. We gather patients’ clinical information related to cancer screening, diagnosis and treatment in the electronic cancer health record (historia clínica electrónica oncológica). This serves as a repository of patient information related to cancer and can be used to share information for the benefit of these patients. We have done many public information campaigns to tell people what they can do to prevent cancer and when they should come to the health clinic for check-ups.

Q: Uruguay is a small country with a population of about 3.4 million. How have you managed to fight the influence of the global tobacco business?

A: The main challenge is to make the general public more aware of why we need tough tobacco control measures and why we need to reduce the risk factors for so many diseases. Another challenge comes from outside our country. The tobacco industry has tried unsuccessfully to make us reverse our tough tobacco control measures. In 2010, tobacco multinational Philip Morris International challenged Uruguay at the World Bank’s international centre for settlement of investment disputes for an alleged breach of the investment protection agreement between Uruguay and Switzerland. Philip Morris International challenged measures related to restrictions on different presentations of the same cigarette brand, the increase in the size of mandatory health warnings on cigarette boxes from 50% to 80% and the use of pictograms with striking images of the health consequences of smoking. The company lost the case in 2016.

Q: How are you protecting your population’s health in spite of globalized food and soft drinks companies and the global alcohol industry?

A: Our approach is to combine public education with tough regulatory action. We have prohibited the sale of specially selected unhealthy foods in schools and colleges. The ministry of public health has published a guide to healthy eating for the general population. We plan to introduce tough requirements for the labelling of highly processed foods to guide the consumer and for the catering sector to offer foods with low or zero salt content. We have several tough alcohol control measures in place, including a zero tolerance law for people who drink alcohol and drive and we plan to extend restrictions on the sale of alcoholic beverages. Finally, we promote physical activity for people of all ages. For example, every school must now have a physical education teacher, we have installed gym equipment in public places and we have created a new post for a minister of sport.

“The WHO Framework Convention on Tobacco Control is the beacon that shows us – all countries – the way in the fight against NCDs.”

Q: You are hosting the Global Conference on NCDs this month. What do you hope the event can achieve?

“Between 65% and 70% of total mortality is attributable to NCDs. In some parts of the world the burden of disease and death due to NCDs is increasing. It’s a real pandemic and one that needs global solutions.”

A: Between 65% and 70% of total mortality is attributable to NCDs. In some parts of the world the burden of disease and death due to NCDs is increasing. It’s a real pandemic and one that needs global solutions. The WHO Framework Convention on Tobacco Control is the beacon that shows us the way in the fight against NCDs. This agreement is based on strong scientific evidence and proposes concrete and effective ways to reduce risk factors to help people become healthier and, ultimately, to save lives. Countries need to comply with the WHO Framework Convention on Tobacco Control so that they can make progress towards attaining sustainable development goal target 3.4 on NCDs by 2030. Uruguay has a tradition of fighting NCDs and, at the conference this month we are keen to find common solutions with other countries for our shared problems. We hope that the Montevideo roadmap, the final outcome of the WHO conference on NCDs this month, will guide and drive countries’ efforts to take action so together we protect the health of our people and save more lives.

